# The Confidence and Accuracy of Medical Students Trained in Ultrasound or Landmarks for Performing Knee Aspiration

**DOI:** 10.7759/cureus.31722

**Published:** 2022-11-21

**Authors:** Emily R German, Katelyn A Hogge, Brooke L Reese, Tom Lindsey

**Affiliations:** 1 Medicine, Edward Via College of Osteopathic Medicine (VCOM) Carolinas, Spartanburg, USA; 2 Surgery, Edward Via College of Osteopathic Medicine (VCOM) Carolinas, Spartanburg, USA

**Keywords:** ultrasound (us), skills and simulation training, orthopedic procedures, medical student training, procedure training, anatomical landmarks, accuracy, trainee confidence, knee aspiration, ultrasound-guided

## Abstract

Introduction

Medical students are trained using anatomical landmarks in order to perform many procedures, such as knee aspirations. With the growing popularity and use of ultrasound, the question arises whether training students with ultrasound instead of landmarks increases their skill. Previous research has shown increased accuracy and confidence in residents who trained with ultrasound compared to landmarks only. No studies to date have analyzed the effect of ultrasound learning versus landmark learning in medical students.

Objective

The purpose of this study is to analyze the confidence and accuracy of medical students when taught knee aspiration using ultrasound training compared to students taught with landmarks only.

Methods

The project was deemed exempt by the Edward Via College of Osteopathic Medicine (VCOM) Institutional Review Board (IRB) because it falls under the quality improvement category (IRB number 1806802-1). Subjects were randomized into two groups: one trained in ultrasound and one trained in landmarks for performing knee aspirations. Both groups were tested for accuracy by the ability to aspirate fluid from the model and the number of attempts; each student was given a maximum of three attempts. Documentation included the number of attempts for each student or “no fluid” for those exceeding three attempts. They were then surveyed for confidence. The study took place in an educational setting at VCOM-Carolinas. A total of 42 first-year medical students participated in this study.

Results

Fisher’s exact test showed statistically significant results for confidence (p<0.001) between the ultrasound group (N=22) and non-ultrasound group (N=20) with a power of 0.285. The accuracy of aspirating fluid was not found to be statistically significant (p=0.4805).

Conclusions

Although no significant differences in the accuracy of knee aspirations at short term were seen, there was a clear improvement in student confidence and perceived capability of the skill. Those students who learned using ultrasound-guided techniques were more confident in their ability to accurately perform the technique when compared to their peers.

## Introduction

The use of ultrasound has vastly improved the practice of medicine. This technology allows healthcare professionals to gain a better understanding of the environment beneath the skin and can help to improve accuracy in procedures such as joint aspirations. Ultrasound can be a valuable tool in the education of medical students and residents.

Medical students report deeper satisfaction and higher confidence in their ability to replicate their skills in a clinical setting when ultrasound simulations are incorporated into their curriculum [1]. When comparing landmark-guided joint arthrocentesis with ultrasound-guided joint arthrocentesis by medical residents in cadaver models, the residents felt far more confident in their abilities and their success of treatment with ultrasound-guided arthrocentesis. The study also suggested that the residents were able to perform the skill in fewer attempts, in less time, and slightly more accurately [2]. The literature shows the potential benefit of ultrasound in the accuracy and confidence of residents, as well as the satisfaction of medical students.

Multiple meta-analyses call for more ultrasound in medical education [3]. Ultrasound-facilitated education may prove beneficial for learning imaging practices, as well as learning simulated techniques [4]. However, the impact of primary education with ultrasound versus landmark-guided in medical students is not currently known.

This study aims to bring the existing data regarding the accuracy and confidence of residents using ultrasound and apply it to medical students. The initial hypothesis is that the use of ultrasound by first-year medical students performing a knee aspiration will improve both their accuracy and confidence.

Currently at Edward Via College of Osteopathic Medicine (VCOM) Carolinas, ultrasound training is not offered in the coursework. However, students have the option to learn ultrasound through extracurricular activities or self-directed learning. Procedural skill education is included in the curriculum, and these skills are often used to practice ultrasound skills. It has not been tested whether procedural skill education augmented with ultrasound initially will impact understanding and ability to perform the skill, as well as student confidence. It was predicted that augmentation with ultrasound guidance would improve both student confidence and accuracy when compared to their peers.

## Materials and methods

Forty-two first-year medical students volunteered to be a part of this study. An email was sent to all first-year medical students at VCOM Carolinas giving them the opportunity to participate if they chose to do so on a voluntary basis. The students were then randomized into either the control (non-ultrasound) or experimental (ultrasound) group. There was no random sampling since the samples themselves volunteered. All three second-year osteopathic medical student (OMS-II) researchers were trained to use ultrasound by a local orthopedic surgeon. The OMS-II researchers were all trained at the same time with the orthopedic surgeon to ensure training was consistent.

Students who were placed into the experimental group received a one-hour ultrasound training session provided by the OMS-II researchers. This session consisted of a brief 10-minute presentation on how to perform an ultrasound-guided knee aspiration followed by hands-on practice with the ultrasound machines and knee models. The presentation touched on the benefits of using ultrasound, materials needed to perform the knee aspiration, indications and contraindications, proper positioning of the patient, and procedure steps. The materials listed in the presentation included betadine, sterile gloves, a sterile drape, lidocaine (or another local anesthetic), a 27-gauge needle for local anesthetic, an 18-20-gauge needle for aspiration, an ultrasound machine with sterile gel, and an ultrasound transducer. For proper positioning, students were taught to place the patient in the supine position with the affected knee slightly flexed using a towel or pillow underneath the patient’s knee to approximately 30 degrees.

**Figure 1:**
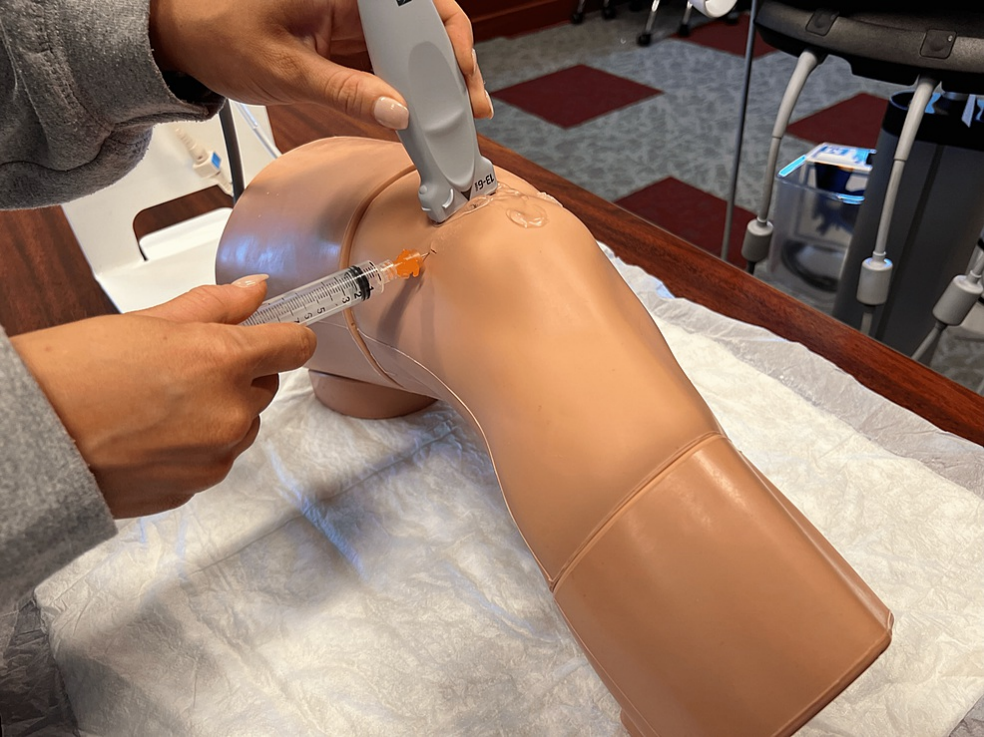
Demonstration of ultrasound-guided knee aspiration

The OMS-II researchers were taught how to perform the knee aspiration in the following eight steps: 1) Place ultrasound gel on the transducer, and find the space supero-lateral to the patella; 2) ensure that the transducer’s long axis is parallel to the needle insertion; 3) cleanse the area on the skin where the needle will be entering; 4) take the needle and anesthetic, enter the skin, and inject the anesthetic; 5) using an 18-gauge needle with a syringe attached, enter the skin keeping parallel to the ultrasound transducer; 6) aspirate the fluid, and send it off; 7) withdraw the needle, and apply pressure to the site; and 8) apply a sterile bandage (Figure 1).

**Figure 2:**
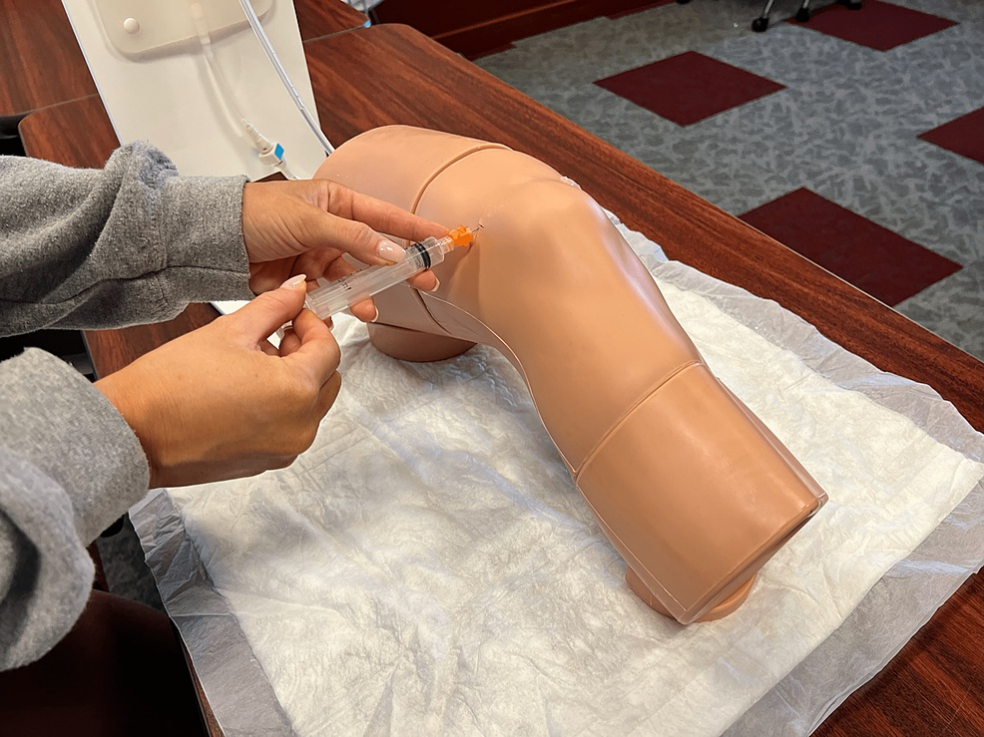
Demonstration of landmark-guided knee aspiration

Students who were placed into the control group did not receive ultrasound training. The control group learned knee aspiration through the VCOM curriculum only, which consisted of landmarking techniques. The landmark was 1 cm above and 1 cm lateral to the superior lateral aspect of the patella at a 45-degree angle (Figure 2).

Following the training of both groups, ultrasound and landmarking, each student was assigned a time to come and completed the skill for an OMS-II researcher in the VCOM Carolinas Simulation Center. All students completed the skill without the use of ultrasound and were given a maximum of three attempts to aspirate fluid from the knee model. If the student was unable to aspirate fluid, this was documented as “no fluid drawn.”

Immediately following the completion of the skill, each student filled out a survey utilizing a Likert scale to assess their confidence in performing the knee aspiration. The survey was conducted using a Google form and consisted of 10 questions (four using Likert scale, four multiple choices, and two open-ended). Within these questions, it was also asked about any previous ultrasound experience to account for any confounding variables. After the completion of the study, the control group was given an opportunity to attend an optional ultrasound training session. This was done to eliminate any unfair advantage. Each question from the survey was analyzed using Fisher’s exact test with a power analysis of 0.285.

This project was reviewed by the VCOM IRB but was deemed to be a quality assessment and therefore was exempt (IRB number 1806802-1). This project did not have funding and did not use a clinical trial registry. Informed consent was implied by request to join the project, as well as completion and submission of the survey. The study was explained in the initial recruitment email, as well as at the beginning of the survey. There was also a statement of informed consent that required a virtual signature before access to the survey was granted. There was no provided compensation to participants, as the data was pre-existing.

## Results

Initial analysis

A total of 42 students were assessed; 20 were in the non-ultrasound group, and 22 were in the ultrasound group. Of the non-ultrasound group, 50% of the students indicated that they have done an aspiration before. For the ultrasound group, 9.1% of students indicated that they had done an aspiration before. The difference in accuracy between groups was found to be insignificant (p=0.4805). In contrast, the perceived confidence level in using the ultrasound machine and in performing joint aspiration using ultrasound was found to be significant (p<0.0001).

Accuracy

Accuracy was tested by the number of attempts to withdraw fluid. Table 1 shows the distribution of the number of attempts for ultrasound and non-ultrasound students. The accuracy of the groups was found to be insignificant with p=0.4805.

**Table 1:** Number of attempts by group

	Ultrasound Group	Non-ultrasound Group	P-value	Statistical Test
Number of attempts	N=22	N=20	0.4805	Fisher’s exact test
1	6 (27.27%)	8 (40.00%)		
2	8 (36.36%)	3 (15.00%)		
3	2 (9.09%)	2 (10.00%)		
No fluid	6 (27.27%)	7 (35.00%)		

Confidence

The confidence of the students was assessed through a survey. Table 2 shows several of the questions asked and the responses of the students by group. The responses were on a five-point scale using the Likert scale. The ultrasound group showed significantly increased confidence in performing the joint aspiration using ultrasound and in their ability to use the ultrasound machine (p<0.0001). They also had significant perception that learning with ultrasound enhanced their learning experience when compared to the non-ultrasound group (p<0.0001). The difference in groups for confidence in performing an aspiration by landmarks was insignificant (p=0.1110).

**Table 2:** Confidence by group *Statistical significance at the α=0.05 level

Survey Questions	Ultrasound Group	Non-ultrasound Group	P-value	Statistical Test
Q1. I feel confident performing a joint aspiration using landmarks	N=22	N=20	0.111	Fisher’s exact test
Strongly disagree	1 (4.55%)	0 (0.00%)		
Disagree	3 (13.64%)	2 (10.00%)		
Neutral	6 (27.27%)	3 (15.00%)		
Agree	7 (31.82%)	14 (70.00%)		
Strongly agree	5 (22.73%)	1 (5.00%)		
Q2. I feel confident performing a joint aspiration using ultrasound	N=22	N=18	<0.0001*	Fisher’s exact test
Strongly disagree	0 (0.00%)	6 (33.33%)		
Disagree	3 (13.64%)	9 (50.00%)		
Neutral	3 (13.64%)	1 (5.56%)		
Agree	11 (50.00%)	1 (5.56%)		
Strongly agree	5 (22.73%)	1 (5.56%)		
Q3. I feel confident in my ability to use the ultrasound machine	N=22	N=17	<0.0001*	Fisher’s exact test
Strongly disagree	0 (0.00%)	5 (29.41%)		
Disagree	2 (9.09%)	10 (58.82%)		
Neutral	3 (13.64%)	1 (5.88%)		
Agree	14 (63.64%)	1 (5.88%)		
Strongly agree	3 (13.64%)	0 (0.00%)		
Q4. I feel that learning with ultrasound enhanced my ability to perform knee aspiration	N=22	N=18	<0.0001*	Fisher’s exact test
Strongly disagree	5 (22.73%)	0 (0.00%)		
Disagree	1 (4.55%)	2 (11.11%)		
Neutral	1 (4.55%)	13 (72.22%)		
Agree	1 (4.55%)	3 (16.67%)		
Strongly agree	14 (63.64%)	0 (0.00%)		
Q5. I feel that learning with ultrasound enhanced my abilities	N=22	N=6	*0.0124	Fisher’s exact test
Only when performing non-ultrasound-guided knee aspiration	0 (0.00%)	2 (33.33%)		
Only when performing ultrasound-guided knee aspiration	7 (31.82%)	3 (50.00%)		
When performing both ultrasound-guided knee aspiration and non-ultrasound-guided knee aspiration	15 (68.18%)	1 (16.67%)		

Additional variables

Several additional variables were looked at to see if they were potential confounders including OMS-II tester, ultrasound versus non-ultrasound training, whether the students had their procedural skills laboratory, and combinations of these. Table 3 shows the additional variables investigated and their p-values. None of these variables were found to be statistically significant.

**Table 3:** Additional variables

Variables as Compared to Score	P-value	Statistical Test
Tester	0.8629	F-test
Ultrasound group versus non-ultrasound group	0.9142	F-test
Skills laboratory: yes or no	0.8893	F-test
Ultrasound group versus non-ultrasound group by tester	0.8456	F-test
Tester by skills laboratory	0.9234	F-test
Ultrasound group versus non-ultrasound group by skills laboratory	0.9581	F-test
Ultrasound group versus non-ultrasound group by skills laboratory and by tester	0.8667	F-test

## Discussion

This project is different from others in that the purpose was to advocate for the augmentation of the currently existing curriculum with ultrasound technology. This was similar to a previous study of medical residents [2], and this project aimed to target the effects on medical students. Another similar study was performed on first-year medical students, which looked at the augmentation of teaching ultrasound for abdominal examinations versus standard instruction. This study showed similar results with increased confidence in ability, although they also showed that delayed use of ultrasound teaching improved ability [5]. This is a possible future investigation for this same model in joint aspiration, in that timing of the teaching may improve ability and confidence.

Studies have shown that medical student anxiety decreases and confidence increases with repeated clinical simulation exposure [6]. This increase in confidence in medical students when performing clinical skills will benefit students upon entering the clinical years of medical training. It has also been shown in students that those who are not confident in their abilities are more hesitant to participate in tasks [7]. The lack of confidence in their ability may lead to students to not be willing to perform clinical procedures given the opportunity.

This examination of different teaching methodologies, although overall successful, had several limitations that appear throughout the course of the project. Despite these limitations, participants in the study demonstrated a positive perspective of the project. Participant confidence was analyzed through the self-reported Likert scale to demonstrate an objective difference between the two groups. The confidence levels reported by the participants showed a statistically significant increase within the ultrasound group on four of five surveyed questions, seen in Table 2. This demonstrates a strong indication that joint aspiration education augmented with ultrasound-guided training may lead to increased confidence in ability among first-year osteopathic medical student (OMS-I) students at VCOM Carolinas.

The model used in this study, teaching medical students clinical skills augmented with ultrasound guidance, is being widely used in academics today. A study that used a similar model to this one concluded that medical students benefit and learn more when using ultrasound to place a peripheral IV compared to the classic landmark-based approach [8]. Because ultrasound provides an imaging modality free of radiation and low costs compared to MRI, it is being used in most clinical settings. The ability to use and understand ultrasound technology in the pre-clinical years of medical training may be viewed as an advanced skill currently but will likely become the standard expectation in the future.

Through the analysis of data validity, several characteristics of the data were utilized to look for potential confounding variables, particularly regarding the OMS-II researchers who performed the ultrasound-guided aspiration education, as well as the evaluations for all 42 subjects. These variables can be seen in Table 3 and were shown to have no statistically significant influence on the data obtained when examined through an F-test. Knee aspiration accuracy was measured by the number of attempts it took the participants to draw fluid or if they were unable to do so within their first three tries. Using Fisher’s exact test, there was no significant difference found between the ultrasound and non-ultrasound groups.

Limitations

Although a statistically significant difference in accuracy was not proven between the two education techniques in the short term, a clear difference in confidence was found to be statistically significant. However, it is important to consider the possibility of several important confounding factors that may have impacted the data collected.

Due to the predetermined skills laboratory schedule, the OMS-I participants all had variable quantities of time between learning knee aspiration and performing the skill for the OMS-II researchers. Times ranged from 30 minutes to about one week between learning the skill and performing it for evaluation, which could have impacted the accuracy of the performed knee aspiration. Nine of the 20 non-ultrasound group participants had previously had their VCOM skills laboratory on joint aspirations prior to their scheduled test day. Data for the ultrasound group was unfortunately unavailable.

Additionally, the OMS-I students had differing levels of background experience regarding knee aspirations, with a total of 16 participants reporting prior relevant experience. Comparing the non-ultrasound group to the ultrasound group, a higher proportion of non-ultrasound group participants indicated that prior relevant experience (nine of 20 participants, 45%, compared to seven of 22 ultrasound group participants, 31.8%) was seen. This could have played a role in the accuracy and confidence of the participants. Additionally, the vast majority of the participants in the ultrasound group, 90.9%, stated they had never performed an aspiration prior to the evaluation. Upon the examination of the non-ultrasound group, only 50% stated that they had never performed an aspiration prior to the evaluation.

An additional limitation of sample size should be acknowledged. There was a limited number of participants in this study, and this will need to be addressed in future studies. The power of the study with groups of 20 and 22 was 0.285. Having a low power reduces the chances of the study demonstrating a true effect. It is generally accepted that a high power would be a value of 0.8 or higher. For future studies using this model, there will need to be enough participants to reach the 0.8 power value.

Future directions

The information gathered has the potential to help launch several follow-up studies. The study will be repeated with new incoming first-year medical students to confirm the results seen. The study will include an increased sample size and an opportunity to minimize confounding factors that arose throughout the completion of the current study. Additionally, the previously examined group of OMS-I participants will be requested for a follow-up study to examine the effect these two education techniques have on the retention of skill accuracy and confidence in the long term. There is also potential to expand the project to multiple VCOM campuses and further increase sample size. Lastly, this study can serve as a prototype for many related questions of how best to augment student education with the current technology available, including the effect of ultrasound-enhanced learning for a variety of other skills such as shoulder aspiration or central line insertion.

## Conclusions

Although the study did not demonstrate significant differences in accuracy for performing a knee aspiration in the short term, the data shows an improvement in student confidence and perceived capability of the skill. Although confidence in ability does not equate to skill, there is educational benefit in students having confidence in their abilities. As technology continues to evolve in clinical medicine, medical students and medical curriculum must be versatile and continue to advance forward with it. Ultrasound education is becoming integral as ultrasound continues to become a highly utilized point-of-care examination. The study hopes to demonstrate the importance of using ultrasound as an augmentation to strengthen students’ learning experience with procedural skills such as joint aspiration.
